# The Constantly Evolving Role of Medical Image Processing in Oncology: From Traditional Medical Image Processing to Imaging Biomarkers and Radiomics

**DOI:** 10.3390/jimaging7080124

**Published:** 2021-07-23

**Authors:** Kostas Marias

**Affiliations:** 1 Department of Electrical and Computer Engineering, Hellenic Mediterranean University, 71410 Heraklion, Greece; kmarias@hmu.gr; 2 Computational Biomedicine Laboratory (CBML), Foundation for Research and Technology—Hellas (FORTH), 70013 Heraklion, Greece

**Keywords:** medical imaging, imaging biomarkers, radiomics, deep learning

## Abstract

The role of medical image computing in oncology is growing stronger, not least due to the unprecedented advancement of computational AI techniques, providing a technological bridge between radiology and oncology, which could significantly accelerate the advancement of precision medicine throughout the cancer care continuum. Medical image processing has been an active field of research for more than three decades, focusing initially on traditional image analysis tasks such as registration segmentation, fusion, and contrast optimization. However, with the advancement of model-based medical image processing, the field of imaging biomarker discovery has focused on transforming functional imaging data into meaningful biomarkers that are able to provide insight into a tumor’s pathophysiology. More recently, the advancement of high-performance computing, in conjunction with the availability of large medical imaging datasets, has enabled the deployment of sophisticated machine learning techniques in the context of radiomics and deep learning modeling. This paper reviews and discusses the evolving role of image analysis and processing through the lens of the abovementioned developments, which hold promise for accelerating precision oncology, in the sense of improved diagnosis, prognosis, and treatment planning of cancer.

## 1. Introduction

To better understand the evolution of medical image processing in oncology, it is necessary to explain the importance of measuring tumor appearance from medical images. Medical image processing approaches contain useful diagnostic and prognostic information that can add precision in cancer care. In addition, because biology is a system of systems, it is reasonable to assume that image-based information may convey multi-level pathophysiology information. This has led to the establishment of many sophisticated predictive and diagnostic image-based biomarker extraction approaches in cancer. In more detail, medical image processing efforts are focused on extracting imaging biomarkers able to decipher the variation within individuals in terms of imaging phenotype, enabling the identification of patient subgroups for precision medicine strategies [1]. From the very beginning, the main prerequisite for clinical use was that quantitative biomarkers must be precise and reproducible. If these conditions are met, imaging biomarkers have the potential to aid clinicians in assessing the pathophysiologic changes in patients and better planning personalized therapy. This is important, as in clinical practice subjective characterizations might be used (e.g., average heterogeneity, speculated mass, necrotic core) which can decrease the precision of diagnostic processes.

Based on the above considerations, the extraction of quantitative parameters characterizing size, shape, texture, and activity can enhance the role of medical imaging in assisting in diagnosis or therapy response assessment. However, in clinical practice, only simpler image metrics (e.g., linear) are often used in oncology, especially in the evaluation of solid tumor response to therapy (e.g., a longer lesion diameter in RECIST). Both RECIST and WHO evaluation criteria rely on anatomical image measurements, mainly in CT or MRI data, and were originally developed mainly for cytotoxic therapies. Such linear measures suffer from high intra/inter-observed variability, which in some cases can compromise the accurate assessment of tumor response, since some studies report inter-observer RECIST variability of up to 30% [2]. Several studies have shown that 3D quantitative response assessments are better correlated with disease progression than those based on 1D linear measurements [3]. Nevertheless, traditional tumor quantification approaches based on linear or 3D tumor measures have experienced substantial difficulties in assessing response to newer oncology therapies, such as targeted, anti-angiogenic treatments and immunotherapies [2]. Size-based tumor assessments do not always represent tumor response to therapy, since, for example, tumors may display internal necrosis formation, with or without reduction in lesion size (as in traditional cytotoxic treatments). Even if RECIST criteria are constantly updated to address these issues, as in the case of Immune RECIST [4], such approaches still do not take into consideration a tumor’s image structure and texture over time. In addition the size and the location of metastases have been reported to play a significant role in assessing early tumor shrinkage and depth of response [5]. To address these limitations, medical image processing has provided over the last few decades the means to extract tumor texture and size descriptors for obtaining more detailed (e.g., pixel-based) descriptors of tissue structure and for discovering feature patterns connected to disease or response. In this paper, it is argued that the evolution of medical image processing has been a gradual process, and the diverse factors that contributed to unprecedented progress in the field with the use of AI are explained. Initially, simplistic approaches to classify benign and malignant masses, e.g., in mammograms, were based on traditional feature extraction and pattern recognition methods. Functional tomographic imaging such as PET gave rise to more sophisticated, model-based approaches from which quantitative markers from tissue properties could be extracted in an effort to optimize diagnosis, treatment stratification, and personalize response criteria. Lastly, the advancement of artificial intelligence enabled the more exhaustive search of imaging phenotype descriptors and led to the increased performance of modern diagnostic and predictive models.

## 2. Traditional Image Analysis: The First Efforts towards CAD Systems

In the 1990s, one of the first challenges in medical image analysis was to facilitate the interpretation of mammograms in the context of national screening programs for breast cancer. In the United Kingdom, the design of the first screening program was undertaken by a working group under Sir Patrick Forrest, whose report was accepted by the government in 1986. As a consequence, the UK screening program was established for women between 50 and 64 in 1990 [6]. The implementation of such screening programs throughout Europe led to the establishment of specialist breast screening centers and the formal training of both radiographers and radiologists. X-ray mammography proved to be a cost-effective imaging modality for national screening, and population screening led to smaller and usually non-palpable masses being increasingly detected.

**Figure 1:**
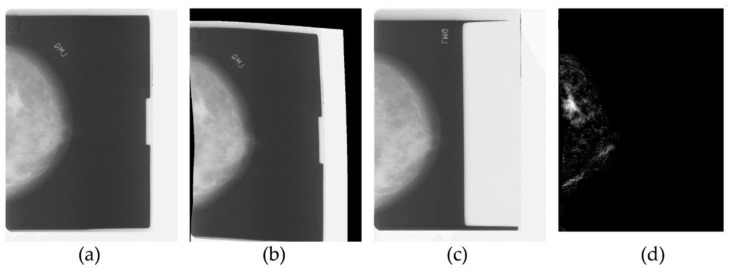
Traditional medical image processing on temporal mammograms. From left to right: the most recent mammogram (**a**) is registered to the previous mammogram (**b**), which is shown in (**c**). After registration there is one predominant region of significant difference in the subtraction image (**d**), which corresponds to a mass developed in the breast.

As a result, the radiologist’s task became more complex, since the interpretation of a mammogram is challenging, due to the projective nature of mammography, while at the same time the need for early and accurate detection of cancer became pressing. To address these needs, medical image analysis became an active field of research in the early nineties, giving rise to numerous research efforts into cancer and microcalcification detection, as well as mammogram registration for improving the comparison of temporal mammograms. Figure 1 depicts the temporal mammogram registration concept towards CAD systems that would facilitate comparison and aid clinicians in early diagnose of cancer in screening mammography [7]. When the ImageChecker system was certified by the FDA for screening mammography in 1998, R2 Technology became the first company to employ computer-assisted diagnosis (CAD) for mammography, and later for digital mammography as well.

However, early diagnostic decision support systems suffered from low precision, which in turn could potentially lead to a negative impact in the number of unnecessary biopsies. In a relevant study [8], the positive predictive values of the interpretations worsened from 100%, 92.7%, and 95.5%, to 86.4%, 97.3%, and 91.1%, when mammograms were analyzed by three independent observers, with and without the CAD. This limitation was representative of the low generalizability of such cancer detection tools in these early days. At the same time the lack of more sophisticated imaging modalities hampered the research efforts towards predicting therapy response and optimizing therapy based on imaging data.

## 3. Quantitative Imaging Based on Models

With the advent of more sophisticated imaging modalities enabling functional imaging, medical image analysis efforts shifted towards the quantification of tissue properties. This opened new horizons in CAD systems towards translating image signals to cancer tissue properties such as perfusion and cellularity and developing more intuitive imaging biomarkers for several cancer imaging applications. For example, in the case of MRI, complex phenomena that occur after excitation are amenable to mathematical modeling, taking into consideration tissue interactions within the tumor microenvironment. In the context of evaluating a model-based approach, the model can be regarded reliable when the predicted data converges on the observed signal intensities and at the same time provides useful insights to radiologists and oncologists. MRI perfusion and diffusion imaging has been the main focus of such modeling efforts, not least due to fact that MRI is ionizing radiation-free.

**Figure 2:**
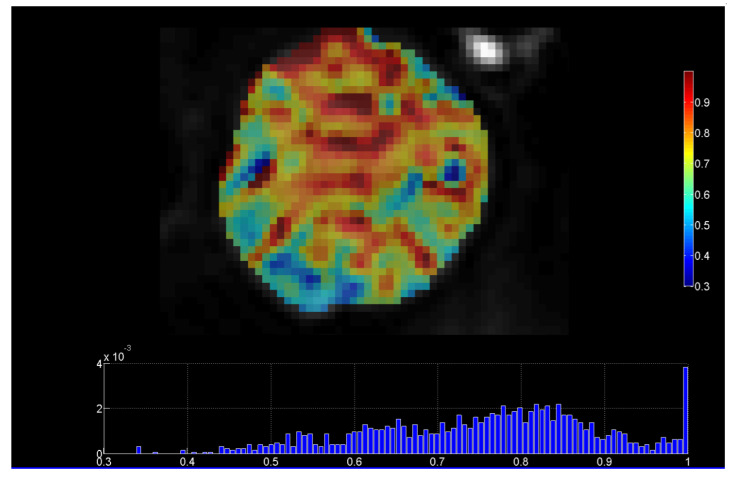
DWI-MRI stretched-exponential (SEM) DWI-MRI parametric map, revealing highly heterogeneous parts of a dedifferentiated liposarcoma (with permission from the department of Medical Imaging, Heraklion University Hospital). Heterogeneity index α ranges from 0 to 1, with lower values of α indicating microstructural heterogeneity.

Diffusion weighted MRI (DWI-MRI) is based on sequences sensitized to microscopic water mobility by means of strong gradient pulses and can provide quantitative information on tumor environment and architecture. Diffusivity can be assessed in the intracellular, extracellular, and intravascular spaces. Apparent diffusion coefficient (ADC) per pixel values derived from DWI-MRI theoretically have an inverse relationship to tumor cell density. In addition, with the introduction of the intravoxel incoherent motion (IVIM) model, both cellularity and microvascular perfusion information could be assessed after parametric modeling [9]. Figure 2 presents a parametric map of the stretching parameter α from the DWI-MRI stretched-exponential model (SEM), revealing highly heterogeneous parts of a dedifferentiated liposarcoma (DDLS) of Grade 3 [9].

DWI-MRI has been tested in most solid tumors for discriminating malignant from benign lesions, to automatize tumor grading, and to predict treatment response and post-treatment monitoring [10].

However, there is still a lack of standardization and generalization of these results, as well as validation against histopathology. While in clinical routine, in-depth DWI-MRI biomarker validation is difficult, recent pre-clinical studies have found that derived parametric maps can serve as a non-invasive marker of cell death and apoptosis in response to treatment [11]. To this end, they also confirmed significant correlations of ADC with immunohistochemistry measurements of cell density, cell death, and apoptosis.

**Figure 3:**
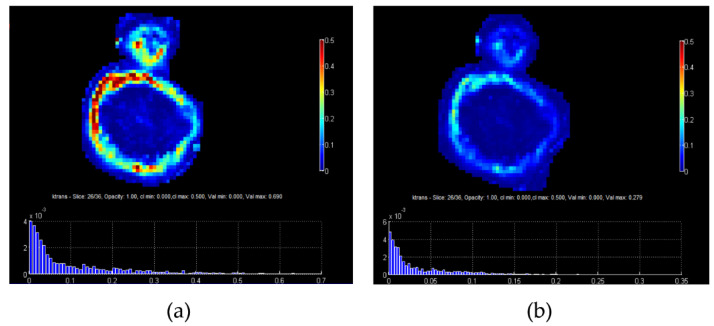
(**a**) ktrans map of a tumor from PK analysis using AIF measured directly from the MR image, while for the conversion from signal to CA concentration the multiple flip angles method (mFAs) was used, (**b**) ktrans map of the same tumor using a population based AIF from Fritz and Hansen.

In a similar fashion, in dynamic contrast-enhanced MRI (DCE-MRI), T1-weighted sequences are acquired before, during, and after the administration of a paramagnetic contrast agent (CA). Tissue-specific information about pathophysiology can be inferred from the dynamics of signal intensity in every pixel of the studied area. Usually this is performed by visual or semi-quantitative analysis from the signal time curves in selected regions of interest. However, with the use of pharmacokinetic modeling, e.g., between the intravascular and the extravascular extracellular space, it became possible to map signal intensities per pixel to CA concentration and then fit model parameters describing, e.g., interstitial space and transfer constant (ktrans). This enabled the generation of parametric maps, e.g., for ktrans providing more quantitative representation of tumor perfusion and heterogeneity within the tumor image region of interest. Although promising, e.g., for assessing treatment efficacy, such approaches have found limited use in clinical practice, not least due to the low reported reproducibility of model parameter estimation. One aspect of this problems is presented in the example shown in Figure 3, where the use of image-driven methods based on multiple-flip angles produces a parametric map of a tumor with different contrast compared to the one produced with the Fritz–Hansen population based AIF [12]. This issue has several implications, including for the accuracy of assessing breast cancer response to neoadjuvant chemotherapy [13].

In conclusion, the clinical translation of DWI and DCE MRI is hampered by low repeatability and reproducibility across several studies in oncology. To address this problem initiatives such as the Quantitative Imaging Biomarkers Alliance (QIBA) propose clinical and technological requirements for quantitative DWI and DCE-derived imaging biomarkers, as well as image acquisition, processing, and quality control recommendations aimed at improving reproducibility error, precision, and accuracy [14]. It is argued that this active area of medical image processing has not yet reached its full potential and still represents a complementary approach to AI driven methods, towards CAD systems for promoting precision oncology. In addition, the exploitation of multimodality imaging strategies (e.g., PET/MRI) can provide added value through the combination of anatomical and functional information.

## 4. Radiomics and Deep Learning Approaches in Oncology through the Cancer Continuum

Traditional cancer medical image analysis was for decades based on human-defined features, usually inspired by low-level image properties, such as intensity, contrast, and a limited number of texture measures. Such methods were successfully used. e.g., in cancer subclassification, but it was hard to capture the high-level, complex patterns that an expert radiologist uses to define the presence or absence of cancer [1].

However, with the advancement of machine learning and the availability of more powerful, high-performance computational infrastructures, it became possible to exhaustively analyze the texture and shape content of medical images in an effort to decipher high-level pathophysiology patterns. At the same time the evolution of texture representation and feature extraction, through a growing number of techniques during the last decades, played a catalytic role in better capturing tumor appearance through medical image analysis [15]. Last but not least, the need to decipher the imaging phenotype in cancer became even more pressing, due to the fact that the vast majority of visible phenotypic variation is now considered attributable to non-genetic determinants in chronic and age-associated disorders [1].

**Figure 4:**
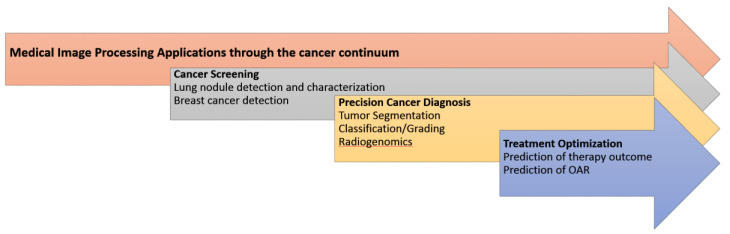
The main medical image processing applications enhanced with AI/radiomics towards precision oncology.

All these factors played a central role in the advancement of radiomics, where in analogy to genomics high-throughput feature extraction followed by ML enabled the development of significant discriminatory and predictive signatures, based on imaging phenotype. Radiomics have been enhanced with deep learning techniques, offering an alternative approach to medical image feature extraction by the learning of complex, high-level features in an automated fashion from a large number of medical images that contain variable instances of a particular tumor. Figure 4 illustrates the main AI/radiomics applications that can assist clinicians in adding precision in the management of cancer patients.

### 4.1. Cancer Screening

Recent advancements in AI driven medical image processing can have a positive impact in national cancer screening programs, alleviating the heavy workload of radiologists and aiding clinicians to reduce the number of missed cancers and to detect them at an earlier stage. Compared to the initial efforts mentioned in previous sections, recent AI-driven image processing can exceed the limits of human vision and potentially reduce the number of cancers missed in screening, as well as cope with inter-observer variability.

Regarding lung cancer screening, early nodule detection and classification is of paramount importance for improving patient outcomes and quality of life. Despite the existence of such screening programs the majority of lung cancers are detected in the later stages, leading to increased mortality and low 5-year survival rate [16]. To this end, radiomics and deep-learning-based methods have shown encouraging results towards precision pulmonary nodule evaluation [17]. A very interesting recent example is reported by Ardill et al., who developed a deep learning algorithm that uses a patient’s current and prior computed tomography volumes to predict the risk of lung cancer. Their model achieved a state-of-the-art performance (94.4% area under the curve) on 6716 cases and performed similarly on an independent clinical validation set of 1139 cases. When prior computed tomography imaging was not available, their model outperformed all six radiologists, with absolute reductions of 11% in false positives and 5% in false negatives [18].

Regarding breast cancer screening technologies, it is argued that AI may provide the means to limit the inherent drawbacks of mammography and enhance diagnostic performance and robustness. In a prospective clinical study, a commercially available AI algorithm was evaluated as an independent reader of screening mammograms, and adequate diagnostic performance was reported [19].

### 4.2. Precision Cancer Diagnosis

During the last decades CAD-driven precision diagnosis has been the holy grail of medical image processing research efforts. However, the clinical interest in such applications has significantly grown only recently with the advancement of AI-driven efforts to generalize performance across diverse datasets. AI systems have reported unprecedented performance regarding the segmentation and classification of cancer. A recent study reported increased performance in segmenting and classifying brain tumors into meningioma, glioma, and pituitary tumors [20].

In addition, a growing number of studies are concerned with automated tumor grading, which is a prerequisite for optimal therapy planning. Yang et al. presented a retrospective glioma grading study (grade II and grade III concerning low grade glioma and high grade glioma) on one hundred and thirteen glioma patients and used transfer learning with AlexNet and GoogLeNet architectures, achieving up to 0.939 AUC [21].

At the same time, the quest to decode imaging phenotype has given rise to efforts to correlate imaging features with molecular and genetic markers in the context of radio-genomics [22]. This promising field of research can provide surrogate molecular information directly from medical images and is not prone to biopsy sampling errors, as the whole tumor can be analyzed. In a recent study, MRI radiomics were able to predict IDH1 mutation with an AUC of up to 90% [23].

### 4.3. Treatment Optimization

There are many challenging problems in optimizing treatment for cancer patients, such as accurate segmentation of organs at risk (OAR) in radiotherapy and prediction of neoadjuvant chemotherapy response. Intelligent processing of medical images has opened new horizons to address these clinical needs. In the case of nasopharyngeal carcinoma radiotherapy planning, a deep learning organs-at-risk (OAR) detection and segmentation network provides useful insights for clinicians for the accurate delineation of OARs [24]. Regarding prediction of neoadjuvant chemotherapy, the use of image-based algorithms to predict outcome has the potential to add precision, not least due to the fact that depending on tumor subtype the outcome can differ significantly. To this end, recent studies report promising preliminary results in applying AI to predict breast cancer neoadjuvant therapy response. Vulchi et al. reported improved prediction of response to HER2-targeted neoadjuvant therapy based on deep learning of DCE-MRI data [25]. Notably, the AUC dropped from 0.93 to 0.85 in the external validation cohort.

## 5. Radiomics Limitations Regarding Clinical Translation

While promising, radiomics methodologies are still in a translational phase and thorough clinical validation is needed towards clinical translation. To this end, when these technologies are tested and reviewed, a number of important limitations becomes apparent. In a recent review on MRI based radiomics in nasopharyngeal cancer [26], the authors reviewed the state of the art and used a radiomic quality score assessment (RQS). Several limitations were highlighted in the reviewed studies, including the absence of a validation cohort in 21% of them, as well as the lack of external validation in 92% of them. In another RQS based evaluation study on radiomics and radio-genomics papers, the RQS was low regarding clinical utility, test-retest analysis, prospective study, and open science [27]. It was also very interesting that no single study used phantoms to assess the robustness of radiomics features or performed a cost-effectiveness analysis. In a similar fashion, lack of feature robustness assessment and external validation was reported in studies regarding prostate cancer [28], while the main reported shortcomings in the quality of the MRI lymphoma radiomics studies regarded inconsistencies in the segmentation process and the lack of temporal data to increase model robustness [29]. All these recent studies clearly indicate that, although medical image processing in oncology has evolved significantly, the clinical translation of radiomics is still hampered by the lack of extensive, high quality validation studies. In addition, the lack of standardization in radiomics extraction remains a problem, which is currently being investigated by several studies, with respect to different software packages [30] and the reproducibility of standardized radiomics features using multi-modality patient data [31].

## 6. Discussion

Contrary to common belief, medical image processing has been evolving for the last few decades and its main application is cancer image analysis. Traditional medical image processing was founded on classical image processing and computer vision principles, focusing on low-level feature extraction and simple classification tasks, e.g., benign vs. malignant, or in the geometrical alignment of temporal images and the segmentation of tumors for volumetric analyses. This early stage in the 1990s was an important milestone for further development, since several radiologists and oncologists understood the future potential and helped in the creation of a multidisciplinary community on medical image analysis and processing. More importantly, it laid the foundations of radiomics by proposing the shape and textural analysis of tumors as useful patterns for detection, segmentation, and classification. However, the main limitation was the high degree of fragmentation in such efforts, the limited computational resources, and the very low availability of cancer image data; usually being mammograms or MRIs.

Functional imaging was another important milestone for medical image computing, since the idea of transforming dynamic image signals to tissue properties paved the way for the discovery of reliable and reproducible image biomarkers for oncology. To achieve this goal, non-conventional medical image processing was deployed based on compartmental models to link the imaging phenotype with microscopic tumor environment properties, based on diffusion and perfusion. Such model-based approaches include compartment pharmacokinetic models for DCE-MRI and the IVIM model for DWI-MRI, often requiring laborious pre-processing to transform the original signal to quantitative parametric maps able to convey perfusion and cellularity information to the clinician. It is argued that this is still an evolving research field and that the potential for clinical translation is significant, especially since techniques such as DWI-MRI do not involve ionizing radiation or the administration of contrast agent. That said, significant standardization efforts are still required in order to converge on stable imaging protocols and model implementations that will guarantee reproducible parametric maps and robust cancer biomarkers. Another limitation when comparing to modern radiomics/deep learning efforts is that the processing of such functional data with compartmental models is a very demanding task, requiring a deeper understanding of imaging protocols, as well as of numerical analysis methods for model fitting.

The gradual advancements of high-performance computing and machine learning and neural networks have revolutionized research in the field, especially during the last decade. The field of radiomics has extended the cancer medical image processing concepts regarding texture and shape descriptors to massive feature extraction and modeling. Such radiomics approaches have also been enhanced by convolutional neural networks, which outperformed the traditional image analysis methods in tasks such as lesion segmentation, while introducing more sophisticated predictive, diagnostic, and correlative pipelines towards precision diagnostics, therapy optimization, and synergistic radio-genomic biomarker discovery. The availability of open access computational tools for machine and deep learning, in combination with public cancer image resources such as the Cancer Imaging Archive (TCIA), has led to an unprecedented number of publications, AI start-ups, and accelerated discussions for the establishment of AI regulatory processes and clinical translation of such technologies. At the same time, the main limitation of these impressive technologies has been their low explainability, which came as a tradeoff for the impressive performances in oncological applications throughout the cancer continuum. Low explainability also contributed to reduced trust in these models, while the vast number of features explored made generalization difficult, especially due to the large variability of image quality and imaging protocols across vendors and clinical sites.

Medical image processing is still evolving and will continue to provide useful tools and methodological concepts for improving cancer image analysis and interpretation. Data science approaches focusing on radiomics have paved the way for accelerating precision oncology [32]. However, most of the efforts to date only use imaging data, which limits the performance of diagnostic and prognostic tools. To this end, novel data integration paradigms, exploiting both imaging and multi-omics data, is a very promising field for future research [33]. Recent studies have started exploring the synergy of deep learning with quantitative parametric maps. In [34], the authors present a deep learning method to predict good responders of locally advanced rectal cancer trained on apparent diffusion coefficient (ADC) parametric scans from different vendors. The fusion of standard imaging representations with parametric maps, as well as integrative diagnostic approaches [35] involving medical image and other cancer related data, hold promise for increasing accuracy and trustworthiness.

## References

[1] Oakden-Rayner L., Carneiro G., Bessen T., Nascimento J.C., Bradley A.P., Palmer L.J. (2017). Precision Radiology: Predicting longevity using feature engineering and deep learning methods in a radiomics framework. Sci. Rep..

[2] Cai W.-L., Hong G.-B. (2018). Quantitative image analysis for evaluation of tumor response in clinical oncology. Chronic Dis. Transl. Med..

[3] Duran R., Chapiro J., Frangakis C., De Lin M., Schlachter T.R., Schernthaner R.E., Wang Z., Savic L.J., Tacher V., Kamel I.R. (2014). Uveal melanoma metastatic to the liver: The role of quantitative volumetric contrast-enhanced MR imaging in the assessment of early tumor response after transarterialchemo. Transl. Oncol..

[4] Aykan N.F., Özatlı T. (2020). Objective response rate assessment in oncology: Current situation and future expectations. World J. Clin. Oncol..

[5] Froelich M.F., Petersen E.L., Heinemann V., Nörenberg D., Hesse N., Gesenhues A.B., Modest D.P., Sommer W.H., Hofmann F.O., Stintzing S. (2020). Impact of Size and Location of Metastases on Early Tumor Shrinkage and Depth of Response in Patients With Metastatic Colorectal Cancer: Subgroup Findings of the Randomized, Open-Label Phase 3 Trial FIRE-3/AIO KRK-0306. Clin. Colorectal Cancer.

[6] Sasieni P. (2003). Evaluation of the UK breast screening programmes. Ann. Oncol..

[7] Marias K., Behrenbruch C., Parbhoo S., Seifalian A., Brady M. (2005). A registration framework for the comparison of mammogram sequences. IEEE Trans. Med. Imaging.

[8] Funovics M., Schamp S., Lackner B., Wunderbaldinger P., Lechner G., Wolf G. (1998). Computerassistierte diagnose in der mammographie: Das R2 imagechecker- system in der detektion spikulierter lasionen. Wien. Med. Wochenschr..

[9] Manikis G.C., Nikiforaki K., Lagoudaki E., de Bree E., Maris T.G., Marias K., Karantanas A.H. (2021). Differentiating low from high-grade soft tissue sarcomas using post-processed imaging parameters derived from multiple DWI models. Eur. J. Radiol..

[10] Messina C., Bignone R., Bruno A., Bruno A., Bruno F., Calandri M., Caruso D., Coppolino P., De Robertis R., Gentili F. (2020). Diffusion-Weighted Imaging in Oncology: An Update. Cancers.

[11] Fliedner F.P., Engel T.B., El-Ali H.H., Hansen A.E., Kjaer A. (2020). Diffusion weighted magnetic resonance imaging (DW-MRI) as a non-invasive, tissue cellularity marker to monitor cancer treatment response. BMC Cancer.

[12] Fritz-Hansen T., Rostrup E., Larsson H.B.W., Søndergaard L., Ring P., Henriksen O. (1996). Measurement of the arterial concentration of Gd-DTPA using MRI: A step toward quantitative perfusion imaging. Magn. Reson. Med..

[13] Woolf D.K., Taylor N.J., Makris A., Tunariu N., Collins D.J., Li S.P., Ah-See M.-L., Beresford M., Padhani A.R. (2016). Arterial input functions in dynamic contrast-enhanced magnetic resonance imaging: Which model performs best when assessing breast cancer response?. Br. J. Radiol..

[14] Shukla-Dave A., Obuchowski N.A., Chenevert T.L., Jambawalikar S., Schwartz L.H., Malyarenko D., Huang W., Noworolski S.M., Young R.J., Shiroishi M.S. (2019). Quantitative imaging biomarkers alliance (QIBA) recommendations for improved precision of DWI and DCE-MRI derived biomarkers in multicenter oncology trials. J. Magn. Reson. Imaging.

[15] Liu L., Chen J., Fieguth P., Zhao G., Chellappa R., Pietikäinen M. (2019). From BoW to CNN: Two Decades of Texture Representation for Texture Classification. Int. J. Comput. Vis..

[16] Svoboda E. (2020). Artificial intelligence is improving the detection of lung cancer. Nature.

[17] Binczyk F., Prazuch W., Bozek P., Polanska J. (2021). Radiomics and artificial intelligence in lung cancer screening. Transl. Lung Cancer Res..

[18] Ardila D., Kiraly A.P., Bharadwaj S., Choi B., Reicher J.J., Peng L., Tse D., Etemadi M., Ye W., Corrado G. (2019). End-to-end lung cancer screening with three-dimensional deep learning on low-dose chest computed tomography. Nat. Med..

[19] Salim M., Wåhlin E., Dembrower K., Azavedo E., Foukakis T., Liu Y., Smith K., Eklund M., Strand F. (2020). External Evaluation of 3 Commercial Artificial Intelligence Algorithms for Independent Assessment of Screening Mammograms. JAMA Oncol..

[20] Díaz-Pernas F.J., Martínez-Zarzuela M., Antón-Rodríguez M., González-Ortega D. (2021). A Deep Learning Approach for Brain Tumor Classification and Segmentation Using a Multiscale Convolutional Neural Network. Healthcare.

[21] Yang Y., Yan L.-F., Zhang X., Han Y., Nan H.-Y., Hu Y.-C., Hu B., Yan S.-L., Zhang J., Cheng D.-L. (2018). Glioma Grading on Conventional MR Images: A Deep Learning Study With Transfer Learning. Front. Neurosci..

[22] Trivizakis E., Papadakis G.Z., Souglakos I., Papanikolaou N., Koumakis L., Spandidos D.A., Tsatsakis A., Karantanas A.H., Marias K. (2020). Artificial intelligence radiogenomics for advancing precision and effectiveness in oncologic care (Review). Int. J. Oncol..

[23] Choi Y., Nam Y., Lee Y.S., Kim J., Ahn K.-J., Jang J., Shin N.-Y., Kim B.-S., Jeon S.-S. (2020). IDH1 mutation prediction using MR-based radiomics in glioblastoma: Comparison between manual and fully automated deep learning-based approach of tumor segmentation. Eur. J. Radiol..

[24] Liang S., Tang F., Huang X., Yang K., Zhong T., Hu R., Liu S., Yuan X., Zhang Y. (2019). Deep-learning-based detection and segmentation of organs at risk in nasopharyngeal carcinoma computed tomographic images for radiotherapy planning. Eur. Radiol..

[25] Vulchi M., El Adoui M., Braman N., Turk P., Etesami M., Drisis S., Plecha D., Benjelloun M., Madabhushi A., Abraham J. (2019). Development and external validation of a deep learning model for predicting response to HER2-targeted neoadjuvant therapy from pretreatment breast MRI. J. Clin. Oncol..

[26] Spadarella G., Calareso G., Garanzini E., Ugga L., Cuocolo A., Cuocolo R. (2021). MRI based radiomics in nasopharyngeal cancer: Systematic review and perspectives using radiomic quality score (RQS) assessment. Eur. J. Radiol..

[27] Park J.E., Kim D., Kim H.S., Park S.Y., Kim J.Y., Cho S.J., Shin J.H., Kim J.H. (2020). Quality of science and reporting of radiomics in oncologic studies: Room for improvement according to radiomics quality score and TRIPOD statement. Eur. Radiol..

[28] Stanzione A., Gambardella M., Cuocolo R., Ponsiglione A., Romeo V., Imbriaco M. (2020). Prostate MRI radiomics: A systematic review and radiomic quality score assessment. Eur. J. Radiol..

[29] Wang H., Zhou Y., Li L., Hou W., Ma X., Tian R. (2020). Current status and quality of radiomics studies in lymphoma: A systematic review. Eur. Radiol..

[30] McNitt-Gray M., Napel S., Jaggi A., Mattonen S.A., Hadjiiski L., Muzi M., Goldgof D., Balagurunathan Y., Pierce L.A., Kinahan P.E. (2020). Standardization in Quantitative Imaging: A Multicenter Comparison of Radiomic Features from Different Software Packages on Digital Reference Objects and Patient Data Sets. Tomography.

[31] Zwanenburg A., Vallières M., Abdalah M.A., Aerts H.J.W.L., Andrearczyk V., Apte A., Ashrafinia S., Bakas S., Beukinga R.J., Boellaard R. (2020). The Image Biomarker Standardization Initiative: Standardized Quantitative Radiomics for High-Throughput Image-based Phenotyping. Radiology.

[32] Capobianco E., Dominietto M. (2020). From Medical Imaging to Radiomics: Role of Data Science for Advancing Precision Health. J. Pers. Med..

[33] Rundo L., Militello C., Vitabile S., Russo G., Sala E., Gilardi M.C. (2019). A Survey on Nature-Inspired Medical Image Analysis: A Step Further in Biomedical Data Integration. Fundam. Inform..

[34] Zhu H.-T., Zhang X.-Y., Shi Y.-J., Li X.-T., Sun Y.-S. (2020). A Deep Learning Model to Predict the Response to Neoadjuvant Chemoradiotherapy by the Pretreatment Apparent Diffusion Coefficient Images of Locally Advanced Rectal Cancer. Front. Oncol..

[35] Chaddad A., Daniel P., Sabri S., Desrosiers C., Abdulkarim B. (2019). Integration of Radiomic and Multi-omic Analyses Predicts Survival of Newly Diagnosed IDH1 Wild-Type Glioblastoma. Cancers.

